# Bacterial Colonization of Silver‐Additive Ventilator Circuit in Patients Receiving Mechanical Ventilation: A Randomized Controlled Trial

**DOI:** 10.1111/crj.70058

**Published:** 2025-03-07

**Authors:** Ke‐Yun Chao, Wei‐Lun Liu, Chao‐Yu Chen, Chia‐Hui Su, Shih‐Hsing Yang, Yu‐Tzu Huang

**Affiliations:** ^1^ Department of Respiratory Therapy, College of Medicine Fu Jen Catholic University New Taipei City Taiwan; ^2^ Cardiovascular and Pulmonary Rehabilitation Center, Fu Jen Catholic University Hospital Fu Jen Catholic University New Taipei City Taiwan; ^3^ Telehealth Center Fu Jen Catholic University New Taipei City Taiwan; ^4^ Artificial Intelligence Development Center Fu Jen Catholic University New Taipei City Taiwan; ^5^ School of Medicine, College of Medicine Fu Jen Catholic University New Taipei City Taiwan; ^6^ Department of Critical Care Medicine, Fu Jen Catholic University Hospital Fu Jen Catholic University New Taipei City Taiwan; ^7^ Department of Respiratory Therapy, Fu Jen Catholic University Hospital Fu Jen Catholic University New Taipei City Taiwan; ^8^ Department of Life Science Fu Jen Catholic University New Taipei City Taiwan; ^9^ Infection Control Center, Taipei Tzu Chi Hospital Buddhist Tzu Chi Medical Foundation New Taipei City Taiwan

**Keywords:** antimicrobial agent, bacterial colonization, infection control, silver‐based ion additive, ventilator circuit, ventilator‐associated pneumonia

## Abstract

**Introduction:**

Mechanical ventilation is a significant risk factor for developing ventilator‐associated pneumonia. Although silver‐coated endotracheal tubes have been shown to reduce the bacterial burden, the efficacy of silver‐based ion additive ventilator circuits in reducing bacterial colonization remains unclear.

**Methods:**

This single‐site, randomized controlled trial compared the incidence of bacterial contamination between a silver‐additive ventilator circuit and a ventilator circuit that did not have a silver additive. Bacterial samples were collected from the inspiratory limb and Y‐adaptor of the circuit and analyzed using culture and identification methods.

**Results:**

Bacterial growth was observed in all samples from the control group and in 93.7% and 81.2% of inspiratory limb and Y‐adaptor samples, respectively, from the study group. The colony counts in the inspiratory limb samples were significantly different between the groups, with a higher proportion of undesirable colony counts in the control group compared with the study group. No significant difference between the groups was observed in the colony counts in the Y‐adaptor samples.

**Conclusion:**

The use of a silver‐additive ventilator circuit may reduce bacterial circuit colonization. However, further research with larger sample sizes and more diverse patient populations is necessary to confirm these findings.

**Trial Registration:** ClinicalTrial.gov: NCT04927806

## Graphical abstract

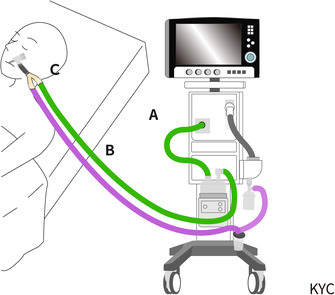

## 1 Introduction

Mechanical ventilation with an endotracheal tube (ETT) is a primary risk factor for ventilator‐associated pneumonia (VAP) [1, 2]. VAP impacts between 5% and 40% of patients undergoing mechanical ventilation for more than 2 days and is a leading cause of morbidity and mortality among patients receiving such ventilation [3, 4, 5, 6, 7, 8, 9, 10]. A comprehensive study conducted by Martin‐Loeches et al. highlights that ventilator‐associated tracheobronchitis presents a substantial global health challenge, correlating with significant resource utilization across various countries [9].

Mechanical ventilation circuits are at risk of bacterial colonization due to the presence of microbes from the patient. Bacterial colonization of a ventilator circuit increases the risk of VAP [11]. Ensuring optimal temperature and humidity conditions for patients with artificial airways is essential. Inappropriate heating and humidification of inspired gas can lead to negative outcomes, including decreased mucociliary clearance, airway mucosal damage, bronchial inflammation, increased respiratory effort, endotracheal occlusion, and prolonged mechanical ventilation [12, 13]. Although active humidification with a heated humidifier (HH) can provide optimal warmth and humidity, condensation in the circuit can create conditions that favor bacterial growth [14, 15]. Contamination of condensation in the ventilator circuit significantly increases the risk of VAP [16]. Therefore, appropriate measures must be taken when using these systems to prevent VAP.

Silver is widely known to be an effective antimicrobial agent and is commonly used to treat infections [17, 18]. Several studies have investigated methods for reducing the incidence of VAP. Studies investigated the effects of intubation with a silver‐coated ETT on the bacterial burden [19, 20]. In several animal studies and clinical trials, silver‐coated ETTs effectively reduced the bacterial burden both on the ETT and in the airway [18, 21, 22, 23].

A company developed an antimicrobial, silver‐based ion additive ventilator circuit [24]. The manufacturer of the device performed antimicrobial testing in accordance with ISO 22196 and reported that the device significantly reduced the viable microbial count levels over time [24]. Although several studies have assessed the efficacy of silver‐coated ETTs [21, 22, 23], no study has investigated the effects of incorporating silver additives into ventilator circuits. Therefore, we conducted a randomized controlled trial to assess the bacterial colonization in a silver‐additive ventilator circuit in an intensive care unit (ICU).

## 2 Methods

This single‐site, open‐label, randomized controlled trial was conducted at Fu Jen Catholic University Hospital, New Taipei City, Taiwan, from November 2021 to May 2022. The study was approved by the Institutional Review Board of Fu Jen Catholic University Hospital (FJUH110126) and was registered with ClinicalTrials.gov (NCT04927806, 07/16/2021). Patients who received mechanical ventilation with an oral ETT at the adult medical ICU were enrolled. Patients who received mechanical ventilation support for fewer than 7 days had an endotracheal or tracheostomy tube in place for more than 24 h prior to their admission to the ICU, did not use the type of ventilator under investigation, or were admitted to a negative pressure room in the ICU were excluded.

**FIGURE 1:**
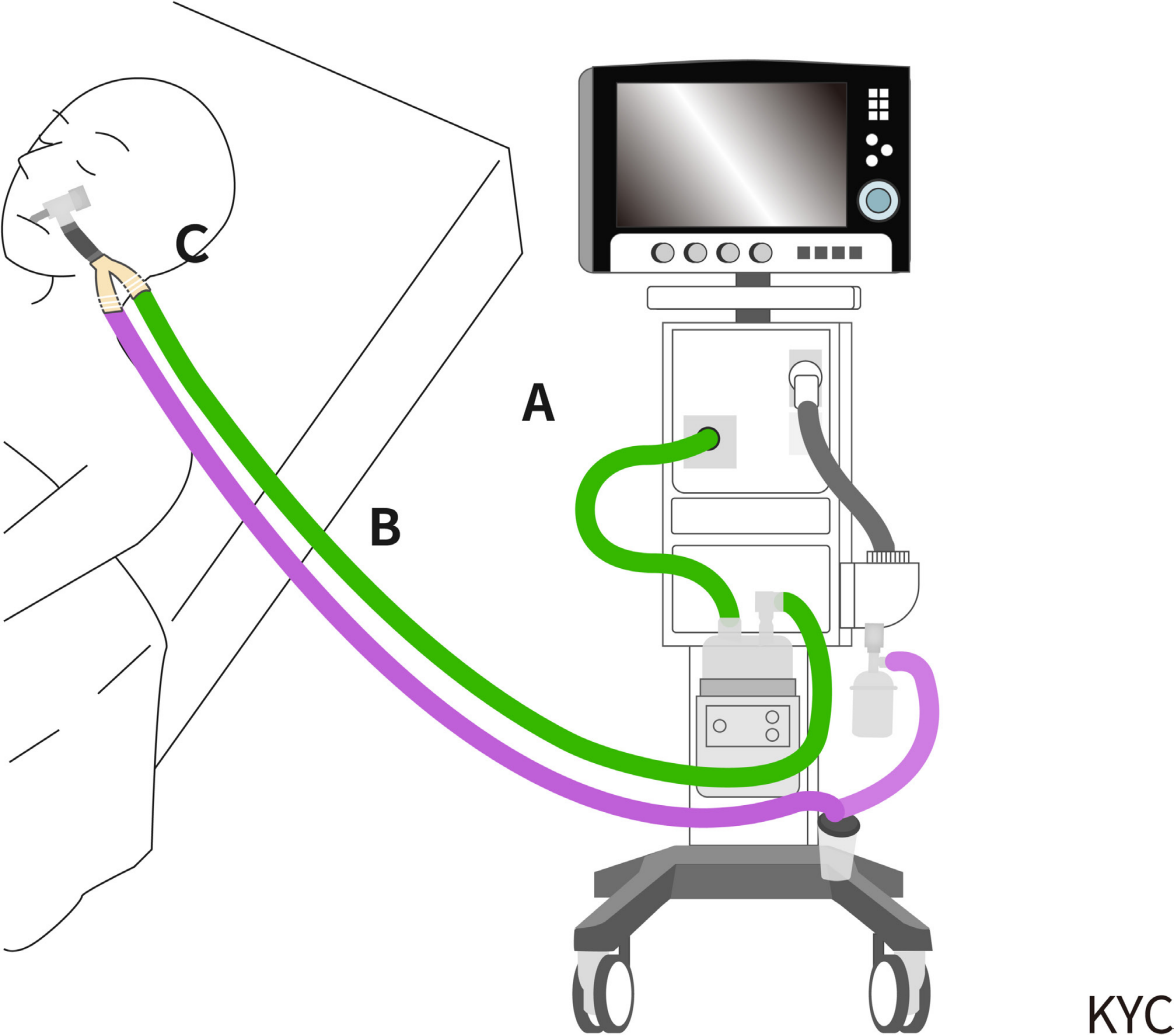
Configuration of ventilator system and sampling site. (A) Inlet tube of heated humidifier. (B) Inspiratory limb. (C) Y‐adaptor. HEPA: high‐efficiency particulate air.

Eligible subjects were allocated into a study or control group according to whether the month of their admission into the ICU was even or odd: The study group received mechanical ventilation with a silver‐additive ventilator circuit and the control group received mechanical ventilation with a ventilator circuit that did not have silver additives. The silver‐additive ventilator circuit was the Silver Knight breathing system (Intersurgical, Wokingham, Berkshire, UK). This device contains silver ions stabilized within a matrix. The ventilator circuit used in the control group was the RT212 ventilator circuit (Fisher & Paykel Healthcare, Auckland, New Zealand). Both ventilator circuits used a closed suction system (T20005, Pacific Hospital Supply, Taipei, Taiwan). To eliminate bias that may be introduced by using different types of ventilators, all samples were collected from the same type of ventilator (Servo‐I, Maquet, Getinge Group Critical Care, Solna, Sweden). An additional high‐efficiency particulate air (HEPA) filter was employed, inserted between the outlet of the ventilator and the inlet of the HH (Figure 1). The ventilator circuit was changed on the seventh day of mechanical ventilation. Samples were collected immediately after the circuit was removed.

The inspiratory limb and Y‐adaptor of the ventilator circuit were selected as the sampling sites (Figure 1). The reference sampling site was the inlet tube of the HH, which was kept clean condition throughout the study period to ensure data reliability.  For Y‐adaptor sampling, a sterile swab moistened with brain heart infusion broth was used. The swab was applied to a 2 × 2 cm^2^ area located 2 cm inside the Y‐adaptor, then inoculated in 5 mL of brain heart infusion broth. The swab was then inoculated in 5‐mL brain heart infusion broth. The inside of the inspiratory limb and the inlet tube of the HH were washed with 40 mL of a sterile tryptone soy broth. All samples were cultured and identified in the Bacteriology Laboratory of Fu Jen Catholic University Hospital. The collected liquid samples were immediately taken 0.5 mL of tryptic soy agar medium for total colony count culture at 35 ± 1°C for 48 ± 2 h, and then the liquid samples were incubated for 16–18 h. After the liquid samples were incubated for 16–18 h, take 50‐μL liquid sample and culture it on Bi‐Blood agar/Eosin Methylene Blue Agar medium. Microbial identification using the VITEK 2XL system (bioMérieux, Marcy l'Etoile, France). The VITEK 2XL is an automated microbiology system utilizing growth‐based technology. Yeast‐like identification was performed using chromogenic Candida agar and cornmeal agar as necessary.

The primary outcome of this study was the incidence of bacterial contamination in the ventilator circuit. The secondary outcome was the distribution of bacterial species in sputum specimens, in the inspiratory limb, and in the Y‐adaptor. Additionally, this study investigated the association between the frequency of circuit disconnection and the level of contamination. The data regarding disconnections was directly retrieved from the ventilator's history log, ensuring the precision and reliability of the information utilized in the analysis. The quantification of bacterial contamination is expressed in terms of colony‐forming units (CFUs), providing a precise measure of bacterial population levels.

Categorical data were expressed as numbers and percentages. Continuous data were presented as medians with interquartile ranges. A Fisher's exact test was used to compare the contamination rates and colony counts between the two groups. Because the sample size was small, a Mann–Whitney U test was used to analyze the continuous variables. Spearman correlation analysis was applied to identify the relationship between the circuit disconnection frequency and contamination for each of the two types of circuits. Statistical analyses were performed in SPSS (version 25.0 for Windows, IBM, Chicago, Illinois, USA). Statistical significance was considered to be indicated by *p* < 0.05.

## 3 Results

**FIGURE 2:**
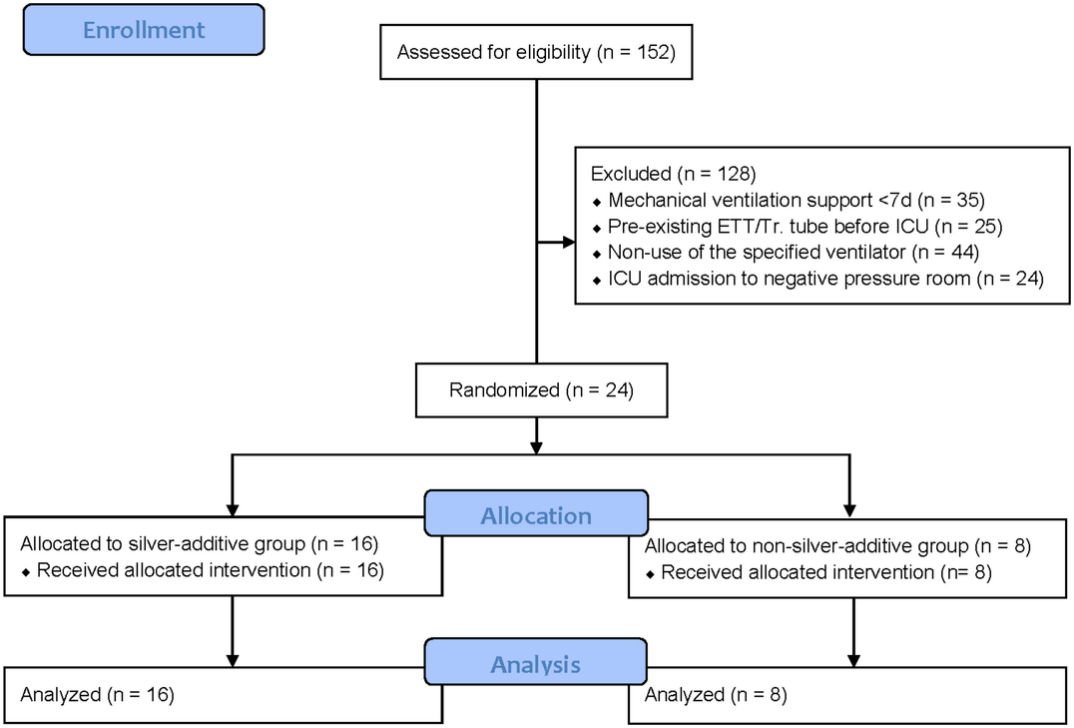
CONSORT flow diagram. ICU: intensive care unit. ETT: endotracheal tube. Tr.: Tracheostomy.

From November 2021 to May 2022, a total of 152 subjects were initially enrolled. However, 128 patients were excluded for the following reasons: 35 received mechanical ventilation for fewer than 7 days, 25 had an endotracheal or tracheostomy tube in place for more than 24 h prior to ICU admission, 44 did not use the specified ventilator, and 24 were admitted to a negative pressure room in the ICU. Ultimately, 24 subjects were enrolled, with 16 in the study group and 8 in the control group. The flow of participants through the study is illustrated in Figure 2. No subjects were lost to follow‐up or excluded from the analysis.

**FIGURE 3:**
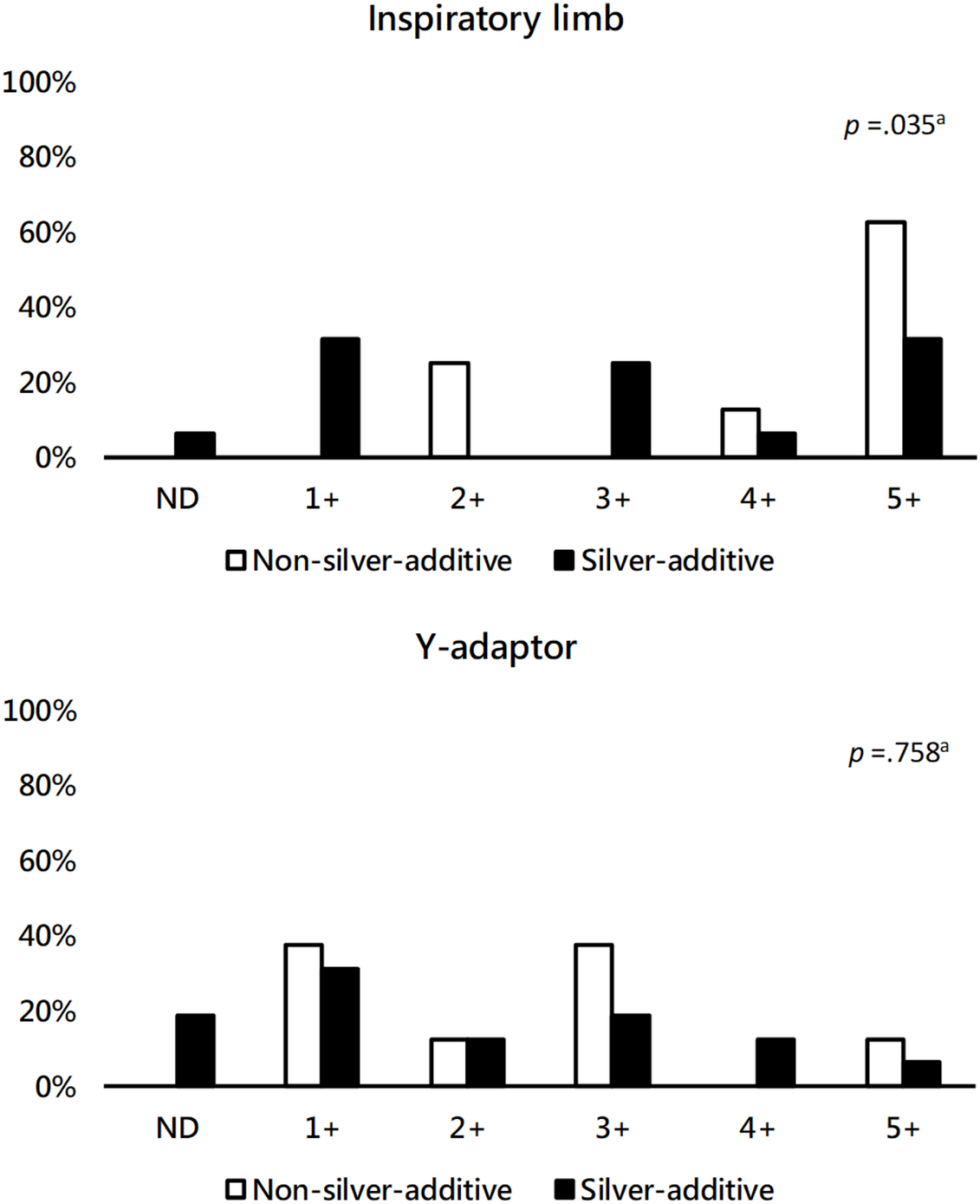
Colony count on inspiratory limb and Y‐adaptor. Data are presented as colony‐forming units (CFUs). ND: not detected. ^a^Fisher's exact test. 1+: 10–1000 CFU. 2+: 1001–10 000 CFU. 3+: 10001–100 000 CFU. 4+: > 100 000 CFU. 5+: Too numerous to count.

The bacterial contamination rates in the inspiratory limb and Y‐adaptor in the study group and control group were similar (*p* > 0.05). In the control group, 100% of the samples had bacterial growth. By contrast, in the study group, 93.7% and 81.2% of the samples from the inspiratory limb and Y‐adaptor, respectively, had bacterial growth; some of the samples in the study group had no bacterial growth (Figure 3). The inlet tube of the HH was determined to be uncontaminated for all study subjects, thereby indicating the credibility of the samples collected in this study. The difference in the inspiratory limb colony counts between the two groups was significant (*p* = 0.035). Specifically, the proportion of inspiratory limb colony counts between 10 and 1000 CFU was higher in the study group (31%) than in the control group (0%). Colony counts within the range of 10–1000 CFU are desirable. The proportion of inspiratory limb colony counts that were too numerous to count was lower in the study group (31.3%) than in the control group (62.5%). No significant difference in Y‐adaptor colony counts was observed between the study and control groups (*p* = 0.758; Figure 3).

The median circuit disconnection frequency in the study group was 34.5 times per 7 days (24.5–43.3 times) and was comparable to that of the control group, which was 19 times per 7 days (15–38.5 times; *p* = 0.07). The correlations between the circuit disconnection frequency and colony count for the whole population (*N* = 24) and the study (*n* = 16) and control (*n* = 8) groups are presented in Table 1. No significant correlation between the circuit disconnection frequency and colony count in the inspiratory limb or Y‐adaptor samples was observed for any of the groups (*p* > 0.05 for all).

**TABLE 1:** Correlation between circuit disconnection frequency and colony count.

Disconnect	Colony count			
	Inspiratory limb		Y‐adaptor	
	*r*	*p*	*r*	*p*
Overall population (*N* = 24)^a^	−0.286	0.175	−0.111	0.607
Non‐silver‐additive (*n* = 8)^a^	−0.397	0.331	0.462	0.249
Silver‐additive (*n* = 16)^a^	−0.259	0.332	−0.271	0.310

^a^ Spearman correlation analysis.

The pathogens identified in the inspiratory limb and Y‐adaptor samples were derived primarily from patient sputum. We compared the bacterial species between the sputum specimens and inspiratory limb and Y‐adaptor samples to assess the efficacy of the silver additive. We cultured the predominant bacteria in the sputum specimens and inspiratory limb and Y‐adaptor samples. The distribution of the bacterial species in the sputum specimens and inspiratory limb samples is presented in Table 2. All isolated pathogens were identified and listed according to risk group. Several of the pathogens detected in the sputum samples were not detected in the inspiratory limb samples in the study group, indicating that the silver‐coated circuit has bactericidal effects. By contrast, in the control group, a similar bactericidal effect was not observed; the pathogens detected in both the sputum and inspiratory limb samples were similar (Table 2).

**TABLE 2:** Pathogens detected in sputum specimens and not detected inspiratory limb samples.

ID	Group	RG	Detected in sputum specimen	Not detected in inspiratory limb samples
1	Study	1	*Candida* species	
		2	*Klebsiella pneumoniae*	*K. pneumoniae*
			*Staphylococcus aureus*	*S. aureus*
			*Staphylococcus warneri*	*S. warneri*
2	Study	1	Yeast‐like	Yeast‐like
		2	*Pseudomonas aeruginosa* (CRPA)	*P. aeruginosa* (CRPA)
3	Study	1	Yeast‐like	Yeast‐like
		2		
4	Study	1	*Candida albicans* *Stenotrophomonas maltophilia*	*C. albicans*
		2	*Acinetobacter baumannii* complex (CRAB) *K. pneumoniae* *P. aeruginosa*	*P. aeruginosa*
5	Study	1	Normal pharyngeal flora	Normal pharyngeal flora
		2	*Haemophilus influenzae*	*H. influenzae*
6	Study	1	*Aspergillus* species	*Aspergillus* species
		2		
7	Study	1	*C. albicans* *S. maltophilia*	*C. albicans*
		2	*Achromobacter xylosoxidans* *Enterococcus faecium* (VRE) *Klebsiella* pneumonia *P. aeruginosa* (CRPA)	*E. faecium* (VRE) *Klebsiella* pneumonia *P. aeruginosa* (CRPA)
8	Study	1	Yeast‐like	Yeast‐like
		2		
9	Study	1	Yeast‐like	Yeast‐like
		2		
10	Study	1		
		2		
11	Study	1	Yeast‐like	Yeast‐like
		2		
12	Study	1	Yeast‐like	Yeast‐like
			Normal pharyngeal flora	Normal pharyngeal flora
		2		
13	Study	1		
		2	*Cryptococcus neoformans*	*C. neoformans*
14	Study	1	Normal pharyngeal flora	Normal pharyngeal flora
		2		
15	Study	1	*C. albicans*	*C. albicans*
		2	*Ralstonia mannitolilytica*	
16	Study	1		
		2	*S. aureus* (MRSA)	*S. aureus* (MRSA)
17	Control	1	*C. albicans*	*C. albicans*
			*S. maltophilia*	*S. maltophilia*
		2	*A. baumannii* complex (CRAB) *Enterobacter aerogenes*	*E. aerogenes*
18	Control	1	*C. albicans*	*C. albicans*
			*Escherichia coli*	*E. coli*
		2	*K. pneumoniae* (CRKP)	*K. pneumoniae* (CRKP)
			*Proteus mirabilis*	*P. mirabilis*
19	Control	1	*C. albicans*	
		2	*Burkholderia cepacia*	
20	Control	1	*S. maltophilia* *Candida tropicalis*	
		2		
21	Control	1	Yeast‐like	Yeast‐like
		2	*R. mannitolilytica*	
22	Control	1	Yeast‐like	Yeast‐like
		2		
23	Control	1	*C. albicans*	*C. albicans*
			*Kocuria rosea*	*K. rosea*
			Yeast‐like	Yeast‐like
		2	*S. aureus* (MRSA)	*S. aureus* (MRSA)
24	Control	1	*C. albicans* Normal pharyngeal flora Yeast‐like	Normal pharyngeal flora Yeast‐like
		2		

Abbreviations: CRAB, Carbapenem‐resistant *Acinetobacter baumannii* ; CRKP, Carbapenem‐resistant *Klebsiella pneumoniae* ; CRPA, Carbapenem‐resistant *Pseudomonas aeruginosa* ; RG, risk group; VRE, Vancomycin‐resistant *Enterococcus*; MRSA, Methicillin‐resistant *Staphylococcus aureus*.

The silver additive was discovered to be effective against bacteria that cause nosocomial infections, including *Staphylococcus aureus* , *Pseudomonas aeruginosa* , and *Stenotrophomonas maltophilia* , and against antibiotic‐resistant bacteria, including methicillin‐resistant *S. aureus* and carbapenem‐resistant *P. aeruginosa* . *Candida* species and yeast‐like pathogens survived in circuits that did not have the silver additive.

## 4 Discussion

To the best of our knowledge, this study represents the first attempt to investigate bacterial contamination of mechanical ventilation circuits that incorporate silver additives. Although extensive research on the use of silver‐coated ETTs has been conducted [25], the efficacy of silver‐based ion additive ventilator circuits in reducing bacterial colonization remains uncertain. Therefore, this study provides novel insights into the efficacy of silver‐additive circuits in reducing bacterial contamination in the context of mechanical ventilation.

Because of the coronavirus disease 2019 (COVID‐19) pandemic, this study experienced a lower‐than‐anticipated enrollment rate. In total, 24 patients who were placed in negative pressure rooms and 44 patients who were not using the ventilator under investigation were excluded. During the study period, almost one‐third of the study hospital's Servo‐I ventilators were allocated to negative pressure rooms for patients with COVID‐19; this may have affected enrollment. Additionally, enrollment had to be terminated in May 2022 for pandemic‐related reasons. The generalizability of the study's findings may be affected by these limitations, and caution is warranted when interpreting the results. Although the sample size was small, which means the statistical power of the results may be insufficient, a lower proportion of inspiratory limb colony counts too numerous to count was observed in the study group compared with the control group. In total, 31.3% of the samples in the study group had colony counts too numerous to count, whereas more than half (62.5%) of the samples in the control group had colony counts too numerous to count. These results indicate that the circuit that did not have a silver additive had a poorer ability to inhibit bacterial growth.

Li et al. [26] conducted a study on bacterial contamination in both reused and disposable ventilator circuits and reported high contamination rates after 1 week of use, with a 100% contamination rate in the inspiratory limb and Y‐adaptor of both types of circuits, which is consistent with our findings. In the present study, in the control group, all samples had bacterial growth, whereas in the study group, only 93.7% and 81.2% of inspiratory limb and Y‐adaptor samples, respectively, had bacterial growth. These results indicate that silver additives may have the potential to reduce bacterial contamination. All ventilator circuits used in the present study were disposable. The ventilator disconnection frequency was comparable between the two groups. Li et al. [26] discovered that ventilator disconnection frequency contributed to bacterial contamination. In the present study, the health‐care workers in our ICU took extra care to minimize the frequency of ventilator circuit disconnections because of the heightened risks associated with COVID‐19 that were present during the study period. Consequently, no significant correlation between disconnection frequency and colony count was observed. Li et al. [26] also discovered that the contamination rate at the inlet tube of the HH was higher in reused circuits (75%) than in disposable circuits (16.7%). Our study provides evidence in support of the benefits of using disposable circuits; we did not detect any bacterial growth at the inlet tube of the HH. The HEPA filter we placed between the ventilator outlet and the HH inlet may have contributed to this result. However, this was a single‐center study, and further studies are warranted to validate our findings.

VAP continues to be a significant risk for patients receiving mechanical ventilation. Numerous care bundles have been developed and implemented to decrease the incidence of VAP [27]. The use of medical supplies containing silver has been proven effective, as evidenced by silver‐coated ETTs. For instance, a larger multicenter trial, the NASCENT trial [20], and a systematic review and meta‐analysis [25] indicated that silver‐coated ETTs successfully reduced the incidence of VAP and delayed the time to VAP occurrence.

Our study suggests that silver‐additive ventilator circuits may similarly play a role in reducing bacterial colonization in ventilator circuits, which is a precursor to VAP. By reducing the bacterial load in the ventilator circuit, the risk of bacterial aspiration and subsequent pneumonia could be lowered. Therefore, integrating silver‐additive ventilator circuits into the care bundle for mechanically ventilated patients might offer an additional measure to prevent VAP. However, it is crucial to conduct further research with larger sample sizes and diverse patient populations to confirm these findings and establish robust clinical guidelines.

Chronic infections resulting from open exposure to the external environment in the ICU are often polymicrobial, involving bacteria–bacteria, bacteria–fungus, and fungi–fungal aggregate interactions [28]. Microorganisms commonly associated with ventilator use include *S. aureus* , *P. aeruginosa* , *Klebsiella pneumoniae* , and *Candida albicans* [29]. We identified these and other drug‐resistant strains. A silver‐additive ventilator circuit that effectively reduces bacterial colonization may be used to manage diverse microbial biofilms. Our research on silver antibacterial medical equipment has provided insights into the diversity of microorganisms and their growth and decline in chronic ventilator circuit infections. This knowledge can be applied for the prevention of VAP.

The present study has several limitations that should be addressed. First, the generalizability of the results may be limited because of the single‐site design. Caution is advised when extrapolating these findings to other settings. Future research should focus on conducting larger, multicenter studies to validate these results and assess the impact on clinical outcomes across different environments. This approach will help ensure that the findings are applicable to a broader patient population and various clinical settings. Second, the small sample size and early termination of the study because of COVID‐19 may have limited the study's statistical power and its ability to detect significant differences between the study and control groups. We made a post hoc analysis to compute the achieved power using G*power 3.1. Based on the sample size collected and bacterial growth of both groups, the power of this study is 0.23. Third, the open‐label design may have introduced bias into the assessment of outcomes, which may affect the validity of the results. Fourth, the study's short duration of 7 days limits its ability to detect the long‐term effects of using a silver‐additive ventilator circuit. Fifth, the study only collected samples from the inspiratory limb and Y‐adaptor of the ventilator circuits and, therefore, may have missed contamination sites such as the ETT or expiratory limb. Caution is advised when interpreting the results because these sites may be significant contributors to ventilator‐associated infections. Last, the study did not assess clinical outcomes, such as ICU length of stay, incidence of VAP, or mortality. As such, the clinical relevance of the findings may be limited. Future studies with larger sample sizes, longer follow‐up periods, and comprehensive assessments of clinical outcomes are required to further explore the potential benefits of a silver‐additive ventilator circuit. As in the clinical practice perspective, the two types of ventilator circuits in this study, whether silver‐additive or non‐silver‐additive, are regularly used in our ICU and have similar costs. This similarity in cost makes the implementation of the silver‐additive circuit feasible.

In conclusion, the present study indicates that the use of a silver‐additive ventilator circuit is feasible and may have a role in reducing bacterial circuit colonization. However, because of the limitations of this study, further research with larger sample sizes and more diverse patient populations is required to confirm these findings.

## Author Contributions

The study was conceived and designed by K.Y.C., W.L.L., C.H.S., and S.H.Y. K.Y.C. and C.Y.C. performed the experimental work and collected the data. Data analysis was performed by K.Y.C. and Y.T.H. The paper was written by K.Y.C., C.Y.C., and Y.T.H. All authors revised the manuscript for important intellectual content and approved the final version.

## Ethics Statement

This study was reviewed and approved by the Institutional Review Board of Fu Jen Catholic University Hospital (FJUH110126) and was registered with ClinicalTrials.gov (NCT04927806, 07/16/2021). Written informed consent for participation was not required for this study in accordance with national legislation and institutional requirements.

## Conflicts of Interest

The authors declare no conflicts of interest.

## Data Availability

The datasets used and analyzed during the current study are available from the corresponding author on reasonable request.
